# [^68^Ga]Ga-DOTA-TOC Synthesis by a Cassette Developer System with [^68^Ga]GaCl_3_ from Cyclotron using Liquid Target: An Italian Experience

**DOI:** 10.2174/0118744710379515250506045145

**Published:** 2025-05-09

**Authors:** Michela Cossandi, Massimo Statuto, Giorgio Biasiotto, Gian Luca Viganò, Luca Camoni, Elena Migliorati, Carlo Rodella, Federica Saiani, Luigi Spiazzi, Francesco Bertagna

**Affiliations:** 1 Nuclear Medicine, ASST Spedali Civili di Brescia, Brescia, Italy;; 2 Highly Specialized Laboratory, ASST Spedali Civili di Brescia, University of Brescia, Brescia, Italy;; 3 Clinical Engineering, ASST Spedali Civili di Brescia, Brescia, Italy;; 4 Health Physics, ASST Spedali Civili, Brescia, Italy;; 5 Nuclear Medicine, ASST Spedali Civili di Brescia and University of Brescia, Brescia, Italy

**Keywords:** Cyclotron, liquid target, gastroenteropancreatic neuroendocrine tumors, [^68^Ga]Ga-DOTA-TOC, generator, positron emission tomography, radiopharmaceutical, european pharmacopeia

## Abstract

**Introduction:**

[^68^Ga-DOTA-D-Phe1-Tyr3]octreotide ([^68^Ga]Ga-DOTA-TOC) is a somatostatin analogue largely used in PET/CT applications for the detection of gastroenteropancreatic neuroendocrine tumors (GEP-NET). Initially, it was obtained using a ^68^Ge/^68^Ga generator. The increasing cost of good manufacturing practice-compliant generators has led to the need to find alternative ways of producing Gallium-68 (^68^Ga). The aim of this work is to show the production optimization of [^68^Ga]Ga-DOTA-TOC *via* cyclotron, derived from three years of experience.

**Methods:**

The production of [^68^Ga]GaCl_3_ *via* the ^68^Zn(p,n)^68^Ga reaction was optimized using a PETtrace 800 cyclotron (equipped with ZnO liquid target) and the synthesis of [^68^Ga]Ga-DOTA-TOC was performed by FASTlab2 developer system according to the Guidelines on Good Radiopharmacy Practice (cGRPP). Quality control process was validated according to the current specific monograph (2482) of the European Pharmacopoeia (Ph. Eur.).

**Results:**

[^68^Ga]Ga-DOTA-TOC was produced in 40 minutes; ten validation batches met the quality criteria expected by the Ph. Eur. The synthesis process has involved many issues due to the use of acidic reagents and related corrosion of some components of cyclotron and developer system, resulting in 12.2% failed syntheses and a target breakdown after 11 months.

**Discussion:**

The main issues, their causes and the strategies used to solve them are reported in the troubleshooting section: thanks to these strategies, the number of failed syntheses has decreased, and today, we have achieved a 0% failure rate.

**Conclusion:**

Liquid target production of [^68^Ga]Ga-DOTA-TOC, once consolidated, instead of ^68^Ge/^68^Ga generator has many advantages.

## 1 INTRODUCTION

In recent years, positron emission tomography/computed tomography (PET/CT) with Gallium68-peptides has become essential in the characterization, staging, and definition of a therapy approach for gastroenteropancreatic neuroendocrine tumors (GEP-NET) with low or intermediate malignancy grades [1]. The diagnosis of neuroendocrine tumors (NET) often occurs late because traditional imaging with [^18^F]-FDG ([^18^F] fluoro-2-deoxy-D-glucose) has limitations, but with the advent of ^68^Ga peptides, such as [^68^Ga-DOTA-D-Phe1-Tyr^3^]octreotide ([^68^Ga]Ga-DOTA-TOC) [2]/[^68^Ga-DOTA-D-Phe1-Tyr^3^]octreotate ([^68^Ga]Ga-DOTA-TATE) [3]/[^68^Ga-DOTA-D-Phe1-1NaI^3^]octreotide ([^68^Ga]Ga-DOTA-NOC) [4], it has became possible to identify the primary tumor, assess the presence of small distant metastases, and guide patients toward targeted therapies [2].

Somatostatin peptides are labeled with Gallium-68, a positron-emitting isotope with a half-life of 67.629 minutes: the positron (β+) produces energy in the form of two coincident 0.511 MeV γ rays [5]. The peptide binding with the ^68^Ga isotope is achieved using the organic compound “1,4,7,10-tetraazacyclododecane-1,4,7,10-tetraacetic acid (DOTA)”, a chelator that forms a stable complex with ^68^Ga. The main peptides conjugated with [^68^Ga]Ga-DOTA are analogs of octreotide, an octapeptide similar to somatostatin that can bind to somatostatin receptors (SSTRs) with greater affinity than the endogenous hormone.

The main ones used in nuclear medicine are:

[^68^Ga]Ga-DOTA-TATE

[^68^Ga]Ga-DOTA-TOC

[^68^Ga]Ga-DOTA-NOC

They differ from each other in their affinity for a specific receptor. In particular: [^68^Ga]Ga-DOTA-TATE has an affinity for SSTR2 (highly expressed in NETs); [^68^Ga]Ga-DOTA-NOC has a good affinity for SSTR3 and SSTR5, while [^68^Ga]Ga-DOTA-TOC has an affinity for tumors that overexpress somatostatin receptors SSTR2 and SSTR5 [6, 7], such as low-grade gastroenteropancreatic neuroendocrine tumors (GEP-NET), pheochromocytomas, and paragangliomas [1].

This work focuses on the implementation of production and quality control of [^68^Ga]Ga-DOTA-TOC at the nuclear medicine department of Spedali Civili (Brescia, IT) in accordance with the Guidelines on Good Radiopharmacy Practice (cGRPP) [8] issued by the Radiopharmacy Committee of the European Association of Nuclear Medicine (EANM) [9].

**Fig. (1):**
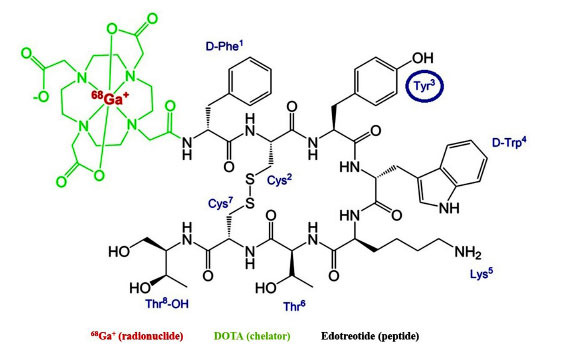
[^68^Ga]Ga-DOTATOC: in green the chelator DOTA, in red Gallium-68 isotope and in black the structure of edotreotide (peptide) in which there is in position 3 a Tyrosine instead of a Phenilalanine. (https://www.mdpi.com/1424-8247/13/3/38).

DOTATOC [DOTA(0)-Phe(1)-Tyr(3)] edotreotide is a peptide with significant utility both in therapy and imaging: it consists of the chelator DOTA covalently linked to edotreotide *via* a peptide bond (the substitution of Phe with Tyr at position 3 increases the stability of the compound) and binds the ^68^Ga radionuclide (as shown in Fig. **1**) [6].

Gallium-68 radionuclide is a largely used isotope for diagnostic applications in nuclear medicine due to its affinity for binding various biomolecular vectors using bifunctional chelators and various macromolecules with rapid pharmacokinetic profiles, such as peptides and peptidomimetics [10].

^68^Ga isotope can be obtained in the form of [^68^Ga]GaCl_3_ from elution of a 1.85 GBq (50mCi) ^68^Ge/^68^Ga generator (Eckert & Ziegler, Germany) using dilute hydrochloric acid (HCl 0.1M - Eckert & Ziegler, Germany). [^68^Ga]GaCl_3_ from the generator is used to label somatostatin analogue peptides by different devices and also to label lyophilized products, thus obtaining [^68^Ga]Ga-DOTA-TOC (edotreotide) for human use [11]. The use of a generator is the only alternative for centers that don’t use a cyclotron. There are many reasons why we have given up using the ^68^Ge/^68^Ga generator, which has led to production of [^68^Ga]GaCl_3_ using a cyclotron: increasingly high costs, 12 months of shelf life of a ^68^Ge/^68^Ga generator, the limited half-life of the ^68^Ge isotope (270.95 days) and the inclusion in the monographs of the European Pharmacopoeia (Ph. Eur.) of Gallium (^68^Ga) chloride (accelerator-produced) solution for radiolabelling (3109), Gallium (^68^Ga) edotreotide injection (2482) and Gallium (^68^Ga) PSMA-11 injection (3044) obtained by cyclotron [12].

[^68^Ga]Ga-PSMA-11 was produced for staging and follow-up of prostate cancers [13] (for which we have a large case series), while [^68^Ga]Ga-DOTA-TOC, as previously mentioned, was used for neuroendocrine tumors; however, in this work we will focus only on the transition of [^68^Ga]Ga-DOTA-TOC production from the ^68^Ge/^68^Ga generator to the cyclotron.

Gallium-68 radionuclide can be obtained by proton irradiation of an enriched Zn^68^ target solution in an accelerator followed by isolation of Gallium-68 in an acidic solution (pH<2.0).

The production of [^68^Ga]GaCl_3_ by cyclotron is possible *via* the ^68^Zn(p,n)^68^Ga reaction using a liquid or a solid target: in this work Gallium-68 was obtained by cyclotron using a liquid target (ZnO 1M in HNO_3_ 0.3M) [14, 15]; a cassette developer system was used for [^68^Ga]GaCl_3_ purification and [^68^Ga]Ga-DOTA-TOC labelling.

Quality control of [^68^Ga]Ga-DOTA-TOC produced was verified in accordance with the current specific monograph (2482) of Ph. Eur., including pH, half-life, radionuclidic purity, chemical identity and radiochemical purity, bacterial endotoxins and sterility [12].

[^68^Ga]Ga-DOTA-TOC synthesis has involved many issues due to the use of acidic reagents and related corrosion of some components of the cyclotron and the developer system: the aim of this work is to solve these problems, derived from an experience of over three years.

## 2 MATERIALS AND METHODS

### 2.1 The Past: Production of [^68^Ga]Ga-DOTA-TOC by ^68^Ge/^68^Ga Generator

[^68^Ga]Ga-DOTATOC was initially produced only by ^68^Ge/^68^Ga generator from May 2018 (month of purchase of the first generator) to December 2020 (a total of 3 generators - 32 months **-** 170 productions).

The 1.85 GBq (50 mCi) ^68^Ge/^68^Ga GalliaPharm Generator (Eckert & Ziegler, Germany) was a GMP-compliant pharmaceutical-grade generator [16]. This generator consisted of a borosilicate glass column containing a bed of titanium dioxide on which ^68^Ge (parent radionuclide, half-life of 270.95 days) was adsorbed: ^68^Ga (daughter radionuclide, half-life of 67.71 minutes) was continuously produced through the decay of its parent and eluted using 0.1 M hydrochloric acid (HCl - Advance Accelerator Applications, Italy), ultra-pure and sterile acid [17]. This system was designed to minimize the breakthrough of ^68^Ge and metallic impurities in accordance with monograph (2464) of the current Ph. Eur. [12]. Activity eluted at the beginning of generator validity was certified to be not less than 1.11GBq and activity eluted at the end of validity (1 year) was certified to be not less than 0.42GBq, as reported in Table **1**.

#### 2.1.1 Labeling Procedure

Radiopharmaceuticals must be prepared by healthcare professionals who have received specific training and have experience in the safe use and handling of radionuclides. Following the Somakit TOC^®^ SmPC (Summary of Product Characteristics), 5 mL of [^68^Ga]GaCl_3_ was eluted through metal-free materials into a Somakit TOC^®^ (40 mg of edotreotide) vial (Advance Accelerator Applications, Italy), [18]; the vial was then placed in a thermoblock at 95°C for 7 minutes. At the end of the process, 0.5 mL of reaction buffer (formic acid, sodium hydroxide, H_2_O - Advance Accelerator Applications, Italy) was added to the vial [17-19]. [^68^Ga]Ga-DOTATOC activity produced was measured using a dose calibrator (COMECER, Italy) and the radiopharmaceutical was verified with quality control before administration to the patient. All these processes were performed following the cGRPP in a class A shielded laminar flow isolator (COMECER, Italy) to ensure the aseptic process and the radioprotection of the operator.

#### 2.1.2 Quality Control Procedure

Somakit TOC^®^ quality control was performed as described in the related SmPC (Summary of Product Characteristics) [17]: appearance (clear solution, free of visible particulates); pH (3.2-3.8) using pH indicator strips (VWR Chemicals, Italy, range 1.0-4.3) radiochemical purity through two thin-layer chromatographies (glass fiber ITLC - Celltech, Italy). The first chromatography used sodium citrate 0.1 M (Sigma Aldrich, USA) as the mobile phase and the second used a mixture of 77g·L^-1^ ammonium acetate (Sigma Aldrich, USA) in H_2_O/methanol (Sigma Aldrich, USA) (50:50 v/v), followed by analysis with a radio-TLC scanner (Elysia-Raytest, Germany) to verify labelling efficiency: presence of colloidal ^68^Ga ≤3%, presence of free ^68^Ga ≤2%.

### 2.2 The Present: Cyclotron production of ^68^Ga, Purification of [^68^Ga]GaCl_3_ and Synthesis of [^68^Ga]Ga-DOTA-TOC

From January 2021 to present, [^68^Ga]Ga-DOTATOC was produced only by cyclotron.

The GE PETtrace 800 series cyclotron (Uppsala, Sweden) for medical use produced radionuclide Gallium-68, a ^68^Ga liquid target with the following characteristics: small target volume (2 mL), water cooling only, 200 mm aluminium degrader foil, stacked niobium (25 mm)/Havar (25 mm) foils. GE PETtrace 800 cyclotron provided a proton beam with a maximum energy of 16.5 MeV and maximum current of 100 mA on target [20]. Preparation of the target solution: Isotopically enriched [^68^Zn]ZnO (665 mg·vial^-1^, ≥ 98.2% enriched - ISOFLEX, San Francisco, California USA - Table **2**) was handled by qualified personnel to prepare [^68^Zn]ZnO 1M in HNO_3_ 0.3M ensuring the use of metal-free materials to avoid compromising the final result [20, 21].

#### 2.2.1 Irradiation of [^68^Zn]ZnO 1M in 0.3M HNO_3_ and Production of ^68^Ga

Direct production of ^68^Ga using a liquid target is based on the reaction ^68^Zn(p,n)^68^Ga: optimization was achieved with a [^68^Zn]ZnO 1M solution in 0.3M HNO_3_ (HNO_3_ 70% ultrapure, Sigma Aldrich, USA) using a 35 μA proton beam for about 60 minutes. During irradiation, two other nuclear reactions occur, which lead to the synthesis of different gallium isotopes, ^66^Zn (p,n)^66^Ga and ^68^Zn (p,2n)^67^Ga, producing ^66^Ga (half-life 9.48 h) and ^67^Ga (half-life 78 h). In particular, the production of ^66^Ga is related to the percentage of ^66^Zn in the isotopically enriched [^68^Zn]ZnO, while the formation of ^67^Ga depends on ^67^Zn in the isotopically enriched [^68^Zn]ZnO and the beam time: increasing the beam time increased the percentage of impurities [21]. In order to prevent cross contamination between the F-18 and Ga-68 production lines, the transfer lines from their respective targets have been completely separated; this way also avoids the possible presence of metal contamination in the Ga-68 production. For optimal ^68^Ga target performance, a conditioning run was completed daily with a short irradiation cycle of 0.6M HNO_3_ to prevent zinc precipitates (proton beam of 15 minutes at a current of 25 μA) [21-23].

#### 2.2.2 [^68^Ga]GaCl_3_ Purification and [^68^Ga]Ga-DOTA-TOC Production

**Fig. (2):**
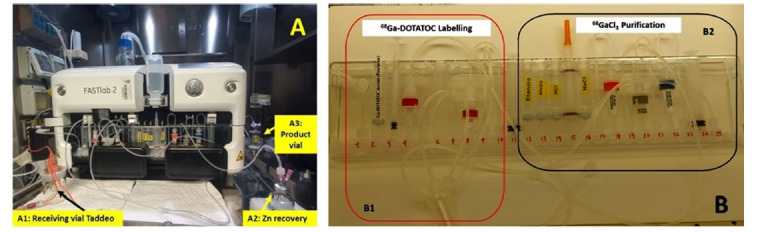
(**A**) Cassette developer system, in particular: (A1) External receiving vial Taddeo, (A2) Zn recovery vial, (A3) Product vial; (**B**) synthesis cassette: the highlighted part on the right (B2) contains the reagents and cartridges for the purification of [^68^Ga]GaCl_3_, while the highlighted part on the left (B1) contains the reagents for the production of [^68^Ga]Ga-DOTATOC.

A cassette developer system (GE HealthCare, Chicago, Illinois, USA) was used, in which [^68^Ga]GaCl_3_ was purified and, sequentially, [^68^Ga]Ga-DOTA-TOC was produced. The same cassette was used for purification and production steps (Fig. **2**); every cassette system was hand-assembled at the moment using the materials reported in Table **3** [24].

[^68^Ga]GaCl_3_ purification step was based on a three-column approach: ^68^Ga solution was transferred into an external receiving vial Taddeo, then into a C18 cartridge to purify contaminants derived from irradiation, such as ^13^N. Subsequently, it was trapped on the first column, a hydroxamate resin (ZR) (previously conditioned with 0.1 M HNO_3_ - Merck Life Science, Italy) for purification of ^68^Ga *via* the irradiation of Zn^68^ on a cyclotron: the zinc was not trapped on the column and residual zinc was washed off the column with 0.1M HNO_3_. ^68^Ga was eluted from ZR resin with HCl 1.75M (Merck Life Science, Italy), passed through a strong anion exchange resin (SAX, previously conditioned with 1.75 M HCl) that removed organic molecules remaining from the manufacturing processes and then trapped onto a third column, a trioctylphosphine oxide, TOPO-based resin (TK200 previously conditioned with 1.75 M HCl), an extractant used in the extraction of various metal ions. This column was washed with 0.1M HCl and 3M NaCl solution: at this step ^68^Ga remained anchored on the column, subsequently, it was eluted from the column into the reactor with water, followed by diluted HCl to formulate [25]. [^68^Ga]GaCl_3_ purified by this system was directly comparable to commercially available ^68^Ge/^68^Ga generators and was compatible with formulations required for pharmaceutical cold kit labeling [23].

[^68^Ga]Ga-DOTA-TOC production step: before the [^68^Ga]GaCl_3_ purification, the reactor was pre-loaded with precursor (50 mg Ga-DOTATOC acetate - ABX, Germany, in a solution of sodium acetate and ascorbic acid - Merck Life Science, Italy); the step of labelling was performed at 95°C for 10 minutes, finally followed a purification step by C18, to yield the [^68^Ga]Ga-DOTA-TOC. The final product (4mL) was filtered using a 0.22mm filter unit (Millex-SG - Merck Life Science, Italy) into a vial containing 8 mL of 10 mM of PBS pH 7.4 (VWR Chemicals, Italy) [24]. [^68^Ga]GaCl_3_ purification and [^68^Ga]Ga-DOTA-TOC synthesis steps took approximately 40 minutes. All these processes were performed in a class A shielded laminar flow isolator (COMECER, Italy) to ensure the aseptic process and the radioprotection of the operator, while the assembly of the components of the cassette and synthesis reagents was prepared in a laminar flow hood placed in a class D environment.

#### 2.2.3 [^68^Ga]Ga-DOTA-TOC Quality Control

Acceptance criteria, specifications and release timing were chosen in compliance with the current general texts and monograph (2482) of the Ph. Eur. [12]. pH was verified using pH indicator strips (VWR Chemicals, Italy, increment 0.5 pH unit); radionuclidic purity by gamma-ray spectrometry (GammaVision - Ortec, Illinois, USA) with principal gamma photons of 0.511 MeV and 1.077 MeV; liquid chromatography was performed on an Ultimate 3000 system (HPLC: High Performance Liquid Chromatography): equipped with a UV variable wavelength detector RS300 (Thermo Fischer Scientific, Germany) and a radiometric detector (GABI, Raytest, Germany). The system was controlled by Chromeleon software version 7.2 SR5 (Dionex Sunnyvale, CA, USA). The column was a base-deactivated end-capped octadecylsilyl silica gel for chromatography (3 μm), 0.15 m - 3.0 mm (Thermo Fisher Scientific, Germany). An isocratic application was performed using a solvent mixture (trifluoroacetic acid from Carlo Erba Reagents, IT/water from Milli-Q Millipore system/ acetonitrile from Sigma Aldrich, USA - 1:780:220 V/V) with a flow rate set at 0.6 mL·min^-1^, UV wavelength at 220 nm, column oven at 25°C. Thin-layer chromatography was performed using a TLC silica gel plate (glass-fibre plate 5x10cm, Merck Life Science, Italy). The solvent for development of TLC plate was a 77g·L^−1^ solution of ammonium acetate in water (Sigma Aldrich, USA), methanol (Sigma Aldrich) (50:50 V/V); plate was developed over 2/3 of its length and was then analyzed with a radio-TLC scanner (Elysia-Raytest, Germany). Determination of residual solvent (ethanol) in the final formulation was carried out by gas chromatography (6850 Series II, Agilent, USA) controlled by Raytest Iberica software (Barcelona, Spain): the column was a GC Column “5cg HP-Fast GC Residual Solvent Column” 30m, 0.53mm, 1.0um (Agilent Technologies, Germany), carrier gas was nitrogen, the flame ionization detector (FID) was set at 300°C and oven temperature was programmed from 33°C to 50°C in 7 min. Bacterial endotoxins were investigated by Endosafe Nexgen-PTS technology (Charles River, Massachusetts, USA) on a 1:40 diluted sample. The sterility instead was verified by an external service (ISZLER: Experimental Zooprophylactic Institute of Lombardia and Emilia-Romagna): the radiopharmaceutical was released for human use before the completion of this test [12].

## 3 RESULTS

Data presented in this section refer to the production and quality control of [^68^Ga]Ga-DOTA-TOC, initially derived from [^68^Ga]GaCl_3_ from a ^68^Ge/^68^Ga generator (170 productions) and later derived from a cyclotron (309 productions). Data were evaluated by descriptive statistical analysis and Student’s t-Test to independent variables.

### 3.1 [^68^Ga]Ga-DOTA-TOC by ^68^Ge/^68^Ga Generator (1.85 Gbq)

Without dwelling too much on this initial data (the aim of this work is focused on the production of [^68^Ga]Ga-DOTA-TOC *via* cyclotron), in Table **4** we show the data of [^68^Ga]Ga-DOTATOC using [^68^Ga]GaCl_3_ derived from a ^68^Ge/^68^Ga generator: specifically, the data related to Generator 1 refer to the year 2018 (from May to December), Generator 2 to the year 2019 (from January to December), and Generator 3 to the year 2020 (from January to December), as production shifted to the cyclotron since 2021. As shown in the table on the data of 3 generators used (years 2018, 2019, 2020), the activity (MBq) of [^68^Ga]DOTATOC ranges from a maximum of 1050 MBq to a minimum of 359 MBq for the first generator, a maximum of 1240 MBq to a minimum of 473 MBq for the second generator, and a maximum of 1340 MBq to a minimum of 457MBq for the third generator. These data comply with the manufacturer's specifications for the generator (Eckert & Ziegler, Germany): activity eluted was a maximum average of 1210 MBq at the beginning of the generator's shelf life and a minimum average of 429 MBq at the end of the shelf life (1 year). Table **5** shows the percentage of failed [^68^Ga]Ga-DOTA-TOC from generator. In 2018, we observed a 2.9%, in 2019, it was around 2.5%, and in 2020 the percentage was 5.3%.

### 3.2 Validation of the [^68^Ga]Ga-DOTA-TOC Production with [^68^Ga]GaCl_3_ from Cyclotron using Target Liquid

The fully automated synthesis of [^68^Ga]Ga-DOTA-TOC using a developer system was completed in 40 min, from the first step of [^68^Ga]GaCl_3_ purification to the [^68^Ga]Ga-DOTA-TOC production step. Ten validation syntheses were performed to verify the critical steps: activity of ^68^Ga trapped in ZR resin and residual activity after purification; activity of [^68^Ga]GaCl_3_ transferred to the reactor for labeling; residual activity in the reactor; activity of [^68^Ga]Ga-DOTA-TOC produced and synthesis yield (Table **6**). The radioactivity sent by the cyclotron at the end of the beam (EOB), at 35mA for 60 minutes, was 3996 Mbq (minimum: 3458 Mbq, maximun: 4502 MBq), the maximum activity produced in the ten validation syntheses of [^68^Ga]Ga-DOTA-TOC was 1725 MBq, and the minimum activity was 1206 MBq (Mean: 1448 MBq), with a decay-corrected yield for decay (time of synthesis: 40 minutes) from a minimum of 37.0% to a maximum of 53.0% (Mean: 44.5%). Table **7** shows the activities of [^68^Ga]Ga-DOTA-TOC obtained after validation’s phase: data refer to the period from 2021 to the first 6 months of 2024 (293 syntheses). As shown, the trend is very consistent and does not experience a negative inflection as it did with the ^68^Ge/^68^Ga generator (due to a short half-life of the generator). As shown in Table **7**, the radioactivity sent by the cyclotron at the end of the beam (EOB), at 35±2 mA for 60 minutes, was a minimum average activity of 4077 MBq and a maximum of 4148 MBq: the minimum average activity produced of [^68^Ga]Ga-DOTA-TOC was 1484 MBq, and the maximum was 1697 MBq (Mean: 1597.5 MBq), with a decay-corrected yield (time of synthesis: 40 minutes) from an average minimum of 45.1% to a maximum of 52.5% (Mean: 48.9%). As can be deduced from this data, which shows the trend broken down by years, there is a linearity in the [^68^Ga]Ga-DOTA-TOC activity produced, which allowed us to ensure doses of 150-200 MBq for 3 patients per production (following the Guidelines of the European Association of Nuclear Medicine - EANM) [26], instead of 2 doses derived from the generator. These results are achieved and consolidated thanks to experience: as detailed in the troubleshooting section, this optimization was possible after three years of work, during which many syntheses failed and several problems related to the use of acids deteriorated the components of the cyclotron and the synthesis module.

### 3.3 [^68^Ga]Ga-DOTA-TOC Quality Control validation

Quality control process was validated for the first 10 syntheses before clinical use: every single production of the validation process, as evidenced in Table **8**, met the requirements set by the current monograph (2482) of the Ph. Eur. [12], so the radiopharmaceutical was approved for human use. The table shows the results of our first 10 validation production batches for clinical use: the radiopharmaceutical appeared clear and colorless; pH fell within the acceptance criteria (3.2-8.0) with an average of 7.05; chemical purity was verified by HPLC (High Performance Liquid Chromatography). Fig. (**3**) shows the HPLC measurement profiles, referring to a reference standard that contains 50 μg·V^−1^ (where V is the maximum injectable volume, in our case equal to 5 mL) of Gallium-edotreotide and 60 μg·V^−1^ of edotreotide. In Fig. (**4**), graphs represent chemical and radiochemical purity: radiochemical purity analyzed in HPLC with a radiometric detector must be at least ≥ 95% of total radioactivity (average of 10 validation synthesis is 97.98%, Table **8**), while to satisfy the requirements of chemical purity, edotreotide and metal complexes of edotreotide analyzed with a UV detector at a wavelength of 200 nm, must not exceed than the area of the peak of Gallium-edotreotide in the standard solution (50 μg·V^−1^), as shown in Fig. (**4**). Ethanol residual solvent, verified in gas chromatography, is < 10% V/V and <2.5 g·dose^−1^ in all 10 syntheses; radionuclidic identity fell within the acceptance criteria (61-75 min) with an average of 66.97 min; the level of bacterial endotoxins is under the limit of 175 IU·V^−1^ and all preparations were sterile. We also verified and validated the stability of the radiopharmaceutical [^68^Ga]Ga-DOTA-TOC: quality control was carried out at time 0 (T0), after 1 hour (T1) and after 2 hours (T2) from the end of synthesis, with particular attention to radiochemical purity, which can diminish over and affect the quality of PET/CT diagnostic images, risking subjecting the patient to ionising radiation without diagnostic benefit. The stability validation was verified up to 2 hours after synthesis: each batch of radiopharmaceutical produced had sufficient activity for 3 patients [26], considering the half-life of ^68^Ga. Here, we present the data from 10 validation radiopharmaceutical preparations, but all 293 preparations were declared suitable for human use, in accordance with the European Pharmacopoeia guidelines.

### 3.4 Syntheses Failed and Troubleshooting Section

**Fig. (5):**
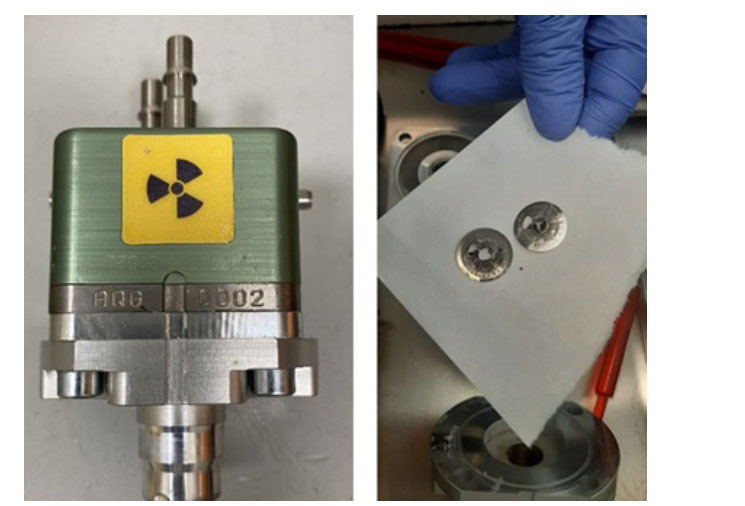
^68^Ga Liquid Target (on the left); use of acids damaged components of gallium liquid target (foils in the figure on the right).

**Fig. (6):**
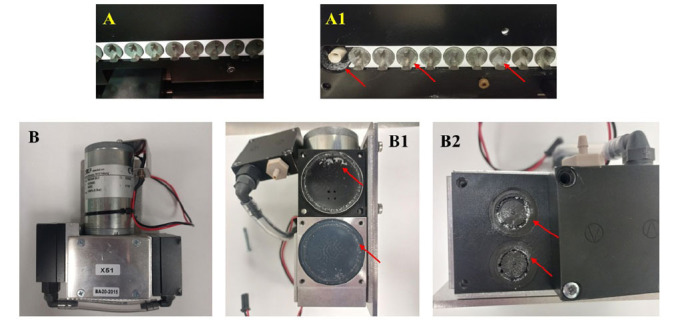
Damaged components of the synthesis module: (**A**) Valve actuators; (**A1**) Valve actuators corroded by acids used for [^68^Ga]GaCl_3_ purification (HCl, HNO_3_). (**B**) Vacuum pump; (**B1** and **B2**) Particular of vacuum pump in which are visible (red arrows) precipitates due to evaporation of HNO_3_.

During the first year of [^68^Ga]Ga-DOTA-TOC production *via* cyclotron, we encountered several problems due to the use of acids, which damaged components of the gallium liquid target (foils and target body) and the synthesis module (Figs. **5** and **6**), resulting in 12.2% failed syntheses (Table **9**) and a target breakdown after 11 months. In Figs. (**5** and **6**), we can see the synthesis module and target parts corroded by acids during the first year of use, which led to the replacement of the entire target and the synthesis module pump. In Table **9** it shows the percentage of failed run: in 2021, we observed a failure rate of 12.2%, in 2022, failed runs decreased to around 3.4%, in 2023, only 2.2% and in 2024 we have not had any failed synthesis. Table **10** reports the main issues in the first period, their causes and the strategies used to solve them. Thanks to these strategies, in the following years, the number of failed syntheses decreased, and today, we have achieved a 0% failure rate. Table **11** shows the data for productions during the first period (68 syntheses, not including test and validation productions) and the second period (225 syntheses): a statistical evaluation was performed on these data by applying a Student’s t-Test to independent variables, assuming that the EOB (End of Beam), EOS (End of synthesis) and yield of synthesis (Table **12**) data from m1 (first period) are lower than the m2 data (second period): the test showed statistical significance with a *p*-value <.001, so the null hypothesis was rejected. From this, it can be concluded that there is no significant difference in activity produced between the first and second period but as already seen in Table **9**, the synthesis process has been consolidated.

## 4 DISCUSSION

Production of [^68^Ga]Ga-DOTATOC (a somatostatin analog used in positron emission tomography/computed tomography, PET/CT, for diagnosis of neuroendocrine tumors) was initially possible, in 2018-2020 (32 months **-** 170 productions) using a ^68^Ge/^68^Ga generator (Eckert & Ziegler, Germany). However, we identified the following negative characteristics of the ^68^Gallium isotope from generator: limited shelf life (1 year), a decrease in activity eluted (a maximum average of 1210 MBq at the beginning of the generator's shelf life and a minimum average of 457 MBq at the end of the shelf life), and the increasingly high costs and dependence on external suppliers. Having a cyclotron already available at the nuclear medicine department of Spedali Civili (Brescia, IT), combined the inclusion in the monograph of the European Pharmacopoeia of [^68^Ga]GaCl_3_ obtained by cyclotron, led us to produce [^68^Ga]Ga-DOTA-TOC by cyclotron.

In our work, we reported data from the production of [^68^Ga]Ga-DOTATOC using a ^68^Ge/^68^Ga generator for human use: as can be seen from Table **4**, the activity eluted gradually decreased in step with the end of the shelf life of the generator (32 months **-** 170 productions).

We described the production, validation and consolidation process, gained from three years of experience (42 months - 309 productions), of the production of [^68^Ga]Ga-DOTA-TOC following the Guidelines on Good Radiopharmacy Practice (cGRPP), and relative quality control, following the current specific monograph (2482) of the Ph. Eur. [12]: ten validation batches of the production process were carried out before human use because, following specific monograph, the preparation may be released for human use before completion of the sterility test. All the productions were sterile (Table **8**), conforming to the safety of the production process.

Referring to the work by Pandey *et al*. 2014 [14] and another by Riga *et al*. 2018 [27] ^68^Ga isotope was produced by cyclotron in a liquid target using a solution of [^68^Zn]ZnO 1M in HNO_3_ 0.2M, after a beam of 60 min at 35mA with a proton energy beam degraded using aluminium foil; the purification of [^68^Ga]GaCl_3_ and subsequent production of [^68^Ga]Ga-DOTA-TOC were carried out using a cassette developer system, preparing reagents in house and manually assembling the synthesis cassette, as described by Rodnick *et al*. 2020 [10]. As can be seen from the results section, the trend in the synthesis of [^68^Ga]Ga-DOTA-TOC by cyclotron remains constant (Table **7**), in contrast to the generator, which has a decreasing trend (Table **4**). The quality control showed chemical, radiochemical purity and radionuclidic identity that met the requirements of the monograph specification (Table **8**). However, as can be seen in the ‘Synthesis failed and troubleshooting section’, the switch to cyclotron production using a liquid target led to many inconveniences due to the use of strong acids and a production failure of around 12.22% in the first year (Table **9**). During the first year, in 2021, we unfortunately experienced target breakdown due to accumulation of [^68^Zn]ZnO precipitates into the target, vacuum pump failure, eluition default of ^68^Ga activity trapped on resin, low activity at EOB (end of beam)or at [^68^Ga]Ga-DOTA-TOC EOS (end of synthesis) and other unforeseen issues (Table **10**).

Some of these problems encountered in the target, mainly due to the use of strong acids, were also described by Z. Ashhar *et al.* [28]; we solved them, or at least mitigated them, by changing the preparation of the 1M ^68^Zn solution to 0.3M HNO_3_ (instead of 0.2M), changing the molarity of the target rinsing solution (HNO_3_ from 0.3M to 0.6M) and leaving it for 60-90 min in the target after a beam of 15 min at 35 mA before transferring it to the receiving vial Taddeo. The increase in HNO_3_ molarity improved the dissolution of ZnO and other metals and avoided the precipitation of metal salts. Moreover, preventive maintenance and reconditioning of the target were conducted every 3500 mA.

For the problems encountered during purification and synthesis, we mitigated them with constant monitoring of the synthesis process and the synthesis module (as described in the appropriate section - Table **10**): constant monitoring of the synthesis module's vacuum pump, checking the synthesis reagents (especially by checking that the nitric acid does not turn yellow), but above all constant updates for strategic resolution.

So, from 12.2% of failed syntheses in 2021, we reduced the failure rate to 3.4% in 2022, 2.2% in 2023 and 0% in the first six months of 2024; 16 failed out of a total of 309 productions (5.2%).

In contrast, in the results section of the productions using a generator, the percentages of failed syntheses are 2.9% in 2018, 2.5% in 2019 and 5.4% in 2020 (Table **5**). As can be seen from the data shown, while in cyclotron production, the number of failed syntheses was successfully reduced by constant monitoring of the target, the synthesis module and revision of the reagent preparation, the same type of monitoring was not possible for generator productions: a minimum of 2.5% of failed labelling is reasonably attributable exclusively to random error of the operator (metal contamination) and/or sudden instrumental (thermoblock) malfunction. The nature of the problems encountered during labelling (with generator and with cyclotron) are totally different, so we didn’t consider it useful to make a comparison, as it’s not meaningful. In this study, we were instead able to monitor the critical points of the synthesis process from the cyclotron and find tricks that made it possible to reduce the failed run percentage.

What we were able to do, instead, was a comparative evaluation of the [^68^Ga]Ga-DOTATOC activity (MBq) produced by generator versus cyclotron: with the generator, we obtained a maximum activity of [^68^Ga]Ga-DOTATOC of 1340 MBq and a minimum of 359 MBq (Table **4**); while with the production of [^68^Ga]Ga-DOTATOC from the cyclotron (Table **7**) a maximum activity of 2514 MBq and a minimum of 950 MBq was seen (with an average synthesis yield corrected for decay of 48.9%). This allowed access to radiopharmaceutical administration for PET/CT imaging evaluation of more patients than the generator: 3 per session instead of 1-2, considering doses of 150-200 MBq [26, 29].

We can therefore claim to have succeeded, thanks to 3 years' experience and 309 syntheses, in optimising and consolidating the production process of [^68^Ga]Ga-DOTATOC, achieving a synthesis yield percentage (corrected for decay) of an average value of 48.9% and an average activity of 1597.5 MBq, while complying with the parameters required by the specific monograph of the European Pharmacopoeia: chemical, radiochemical purity and radionuclidic identity; pH; sterility and pyrogenicity.

## LIMITATION OF THE STUDY

In conclusion, while the study demonstrates that cyclotron production of [^68^Ga]Ga-DOTATOC using a liquid target is an efficient alternative to a generator, economic evaluations will be essential to establish the advantages of this production approach.

## CONCLUSION

The aim of this study was to switch from generator to cyclotron labelling of [^68^Ga]Ga-DOTATOC, ensuring the same radiopharmaceutical quality, safety and efficacy, meeting the requirements of EANM guideline for radiopharmaceuticals, of Summary of Product Characteristics (for generator) and European Pharmacopoeia (for cyclotron).

Liquid target production of ^68^Ga radiopharmaceuticals, instead of the ^68^Ge/^68^Ga generator, is viable only for nuclear medicine centers with a cyclotron: at Spedali Civili (Brescia, Italy), it required the purchase of a target for ^68^Ga, a dedicated synthesis module, and staff training. During the first year of production, we encountered several problems due to the corrosion of cyclotron components and the synthesis module, but optimization of the preparation of the target solution and its washing, as well as constant monitoring of the synthesis module’s vacuum pump has led to excellent results. The advantages of [^68^Ga]Ga-DOTATOC production compared to the generator include: a consistently constant activity production, not affected by the short life of the generator, with the possibility of preparing doses for 3 patients (150-200 MBq for each one) per cyclotron run instead of 2 patients per generator production. Importantly the ability, once [^68^Ga]GaCl_3_ has been purified, to produce new radiopharmaceuticals indipendent of the availability of commercial kits represents a key advantage. We have also validated the synthesis process of [^68^Ga]Ga-PSMA-11 [30-31] and, in the future, we could produce more ^68^Ga-peptides [32-34].

## Figures and Tables

**Fig. (3):**
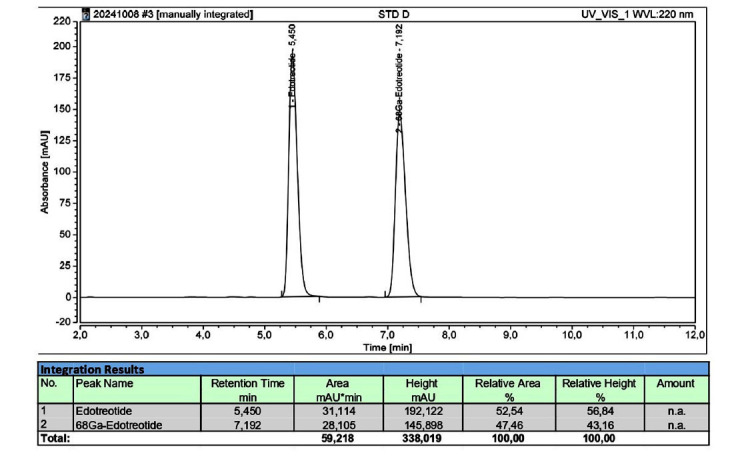
The figure shows the profile of measure of Reference solution verified by isocratic HPLC-Ultimate 3000: 50 mg·V^−1^ Ga-edotreotide and 60 mg·V^−1^ edotreotide.

**Fig. (4):**
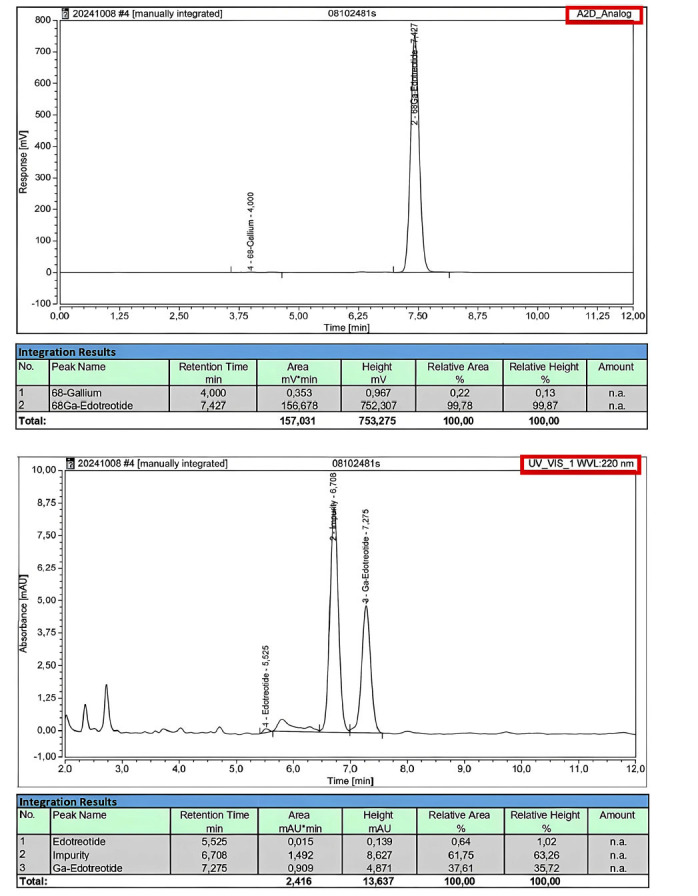
The figures show the profiles of measures obtained by isocratict HPLC-Ultimate 3000, in particular: on the left [^68^Ga]Ga-DOTATOC analized with a radioactivity detector; on the right [^68^Ga]Ga-DOTATOC analized with UV detector.

**Table 1:** Activity on generator and activity from elution. Shelf life of the generator: 1 year.

**Activity (GBq)**	**Activity (GBq) Inside of Generator at the Beginning of Validity**	**Activity (GBq) Inside of Generator at the End of Validity**	**Activity (GBq) Eluted at the Beginning of Validity**	**Activity (GBq) Eluted at the End of Validity**
1,85	1,85 ± 10%	0,7 ± 10%	NLT * 1,11	NLT* 0,42

**Note: ** *NLT (not less than).

**Table 2:** Physical characteristics of [^68^Zn]ZnO (ISOFLEX, San Francisco, California USA).

**Description**		**Isotopic Distribution**		**Chemical Admixtures**			
**Isotope**	**Zn-68**	**Isotope**	**Content (%)**	**Element**	**Content (ppm)**	**Element**	**Content (ppm)**
Enrichment	98.2%	Zn-64	0.1	Al	<1.0	Mg	0.25
Element weight	665 mg	Zn-66	0.180	As	<1.0	Mn	<0.1
Form	Oxide (ZnO)	Zn-67	0.963	Ca	6.6	Pb	2.1
-	-	Zn-68	98.2	Cd	<0.1	Si	6.8
-	-	Zn-70	0.557	Co	<0.1	Sn	53
-	-	-	-	Cu	1.5	Na	<1.0
-				Fe	<1.0	-	-

**Note: ** Data from Certificate of Analysis of [^68^Zn]ZnO (ISOFLEX).

**Table 3:** List of consumables (on the left column) and reagents (on the right column) for developer system.

**Consumables Ready to Use, Handly Assembled on the Developer System**	**Reagents Prepared in the Laboratory using Metal-Free Materials**
• Developer system (GE HealthCare, Chicago, Illinois USA): cassette cover, scheleton and tubing system, reactor, spike for water bag and vials for reagents	➢ 3.1 mL vial of ethanol (ultrapure, Merck Life Science)
• Water bag (BBraun for injecting): store at 4°C to ensure efficient C18 purification	➢ 4 mL vial of HNO_3_ 0.6M (HNO_3_ 70%, ultrapure, Sigma Aldrich)
• 2 C18 cartridges (Waters)	➢ 4 mL vial of HCl 4M (HCl 30%, ultrapure, Merck Life Science)
• 1 ZR resin, 2 mL (hydroxamate-based resin, Triskem) • 1 TK200 resin, 2 mL (trioctylphosphine oxide, TOPO-based resin, Triskem) • 1 SAX resin, 1 mL (strong anion exchange resin, Triskem)	➢ 4 mL vial of NaCl 3M (NaCl, ultrapure, Merck Life Science)
• 1 external vial receiving Taddeo (Comecer)	➢ 1 vial precursor containing: 50mg Ga-DOTATOC acetate (ABX, Advanced Biochemical Compound) dissolved in 1.2 mL solution of sodium acetate (1M, pH 4.5, Merck Life Science) and 100 mL of ascorbic acid (ultrapure, Merck Life Science)

**Table 4:** Activity of [^68^Ga]Ga-DOTATOC labelling with ^68^GaCl_3_ from ^68^Ge/ ^68^Ga generator.

**-**	**Generator 1 ^68^Ga-DOTATOC**	**Generator 2 ^68^Ga-DOTATOC**	**Generator 3 ^68^Ga-DOTATOC**
n	33	78	53
Mean (MBq)	686	913	902
Standard deviation (%)	23.04	21.14	24.61
Minimum (MBq)	359	473	457
Maximum (MBq)	1050	1240	1340

**Note: ** use of the first generator started on May 2018.

**Table 5:** Percentage of failed [^68^Ga]Ga-DOTATOC labelling during the years (2018-2020).

**Year**	**^68^Ga-DOTATOC Labelling**			**Total**
	**Failed**	**Completed**	**% Failed**	
2018	1	33	2.9	34
2019	2	78	2.5	80
2020	3	53	5.4	56
Total	6	164	3.5	170

**Note: ** use of the first generator started on May 2018.

**Table 6:** EOB, ^68^Ga trapped and residual in ZR cartridge, ^68^GaCl_3_ transferred and residual into the reactor, activity and yield of [^68^Ga]Ga-DOTATOC produced at EOS. Time of synthesis: 40 min. Data related to the 10 validation syntheses of the process.

**-**	**^68^Ga EOB (MBq) 35 mA 60 min**	**^68^GaoZR Trapped (MBq)**	**^68^Ga3ZR Residual (MBq)**	**^68^GaCl_3_ Reactor Start Label. (MBq)**	**^68^GaCl_3_ Reactor residual. (MBq)**	**EOS ^68^Ga DOTA-TOC (MBq)**	**Yield (%)**	**Decay Corrected Yield (%)**
Mean	3996	3610	476	2424	1098	1448	35.3	44.5
Minimum	3458	3124	176	2105	820	1206	29.4	37.0
Maximum	4502	4067	923	2813	1500	1725	42.0	53.0

**Table 7:** Data of 293 [^68^Ga]Ga-DOTATOC syntheses (year 2021- first 6 months of 2024).

**-**	**Year**	**^68^Ga EOB (MBq) 35 mA 60 min**	**^68^GaCl_3_ into Reactor (MBq)**	**EOS ^68^Ga DOTA-TOC (MBq)**	**Yield (%)**	**Decay Corrected Yield (%)**
Mean	2021	4135	2311	1515	36.6	46.2
-	2022	4077	2376	1697	41.7	52.5
-	2023	4108	2461	1694	41.2	52.0
-	2024	4148	2211	1484	35.8	45.1
Minimum	2021	4100	1519	950	22.9	28.9
-	2022	3515	1503	1166	28.1	35.5
-	2023	3330	1776	1218	29.1	36.7
-	2024	4107	1720	1019	24.6	31.0
Maximum	2021	4144	3417	1893	45.7	57.6
-	2022	4181	3307	2010	53.8	67.9
-	2023	4292	4303	2514	56.3	70.9
-	2024	4366	4319	2500	52.4	66.0

**Table 8:** Results of [^68^Ga]Ga-DOTATOC quality control (10 validation syntheses).

**No. Synthesis**	**Appearance**	**pH**	**Radiochem. Purity (HPLC)**	**Radiochem. Purity (TLC)**	**Ethanol Residual (GC)**	**Radionuclidic Identity (T1/2)**	**Radionucl. Identity - Gray Spectrometer**	**Endotoxins**	**Sterility Test**
1.	Clear and colorless	7.0	98.88	0.97	<10% V/V <2.5 g·dose^−1^	64.61	√	<175 IU·V^−1^	Sterile
2.	Clear and colorless	7.0	98.41	1.58	<10% V/V <2.5 g·dose^−1^	66.86	√	<175 IU·V^−1^	Sterile
3.	Clear and colorless	7.0	98.88	1.10	<10% V/V <2.5 g·dose^−1^	66.20	√	<175 IU·V^−1^	Sterile
4.	Clear and colorless	7.0	97.76	2.21	<10% V/V <2.5 g·dose^−1^	67.70	√	<175 IU·V^−1^	Sterile
5.	Clear and colorless	7.5	98.24	1.67	<10% V/V <2.5 g·dose^−1^	66.17	√	<175 IU·V^−1^	Sterile
6.	Clear and colorless	7.0	96.53	2.81	<10% V/V <2.5 g·dose^−1^	65.84	√	<175 IU·V^−1^	Sterile
7.	Clear and colorless	7.0	97.05	2.84	<10% V/V <2.5 g·dose^−1^	67.13	√	<175 IU·V^−1^	Sterile
8.	Clear and colorless	7.0	98.12	1.87	<10% V/V <2.5 g·dose^−1^	71.26	√	<175 IU·V^−1^	Sterile
9.	Clear and colorless	7.0	98.32	1.44	<10% V/V <2.5 g·dose^−1^	66.25	√	<175 IU·V^−1^	Sterile
10.	Clear and colorless	7.0	97.51	2.76	<10% V/V <2.5 g·dose^−1^	67.86	√	<175 IU·V^−1^	Sterile
Mean	Clear and colorless	7.05	97.98	1.99	<10% V/V <2.5 g·dose^−1^	66.97	√	<175 IU·V^−1^	Sterile
Acceptance Criteria	Clear and colorless	3.2-8.0	≥ 95%	≤3%	<10% V/V <2.5 g·dose^−1^	61-75 min	Princ. Photons: 0.511 MeV, 1.077 MeV	<175 IU·V^−1^	Sterile

**Table 9:** Percentage of failed [^68^Ga]Ga-DOTATOC production during the years (2021- first six months of 2024).

**Year**	**Syntheses**			**Total**
	**Failed**	**Completed**	**% Failed**	
2021	11	79	12.2	90
2022	3	86	3.4	89
2023	2	87	2.2	89
2024	0	41	0	41
Total	16	293	5.2	309

**Table 10:** Issues encountered in production of ^68^Ga-DOTATOC, possible causes and related troubleshooting.

**Event**	**Possible Causes**	**Troubleshooting**
^68^Ga-Target Breakdown	Accumulation of [^68^Zn]ZnO precipitates into the target	Review in preparation of [^68^Zn]ZnO solution and Cleaning Solution (HNO_3_): 1. Target Solution: [^68^Zn]ZnO 1M in HNO_3_ 0.3M instead of 0.2M (referring to the first period) 2. Cleaning Solution: HNO_3_ 0.6M instead of 0.3 (referring to the first period) 3. Incubation of Cleaning solution into the target for 60-90 minutes, after a beam (15 min at 35 mA), before transfer it to receiving vial Taddeo
Beam drawback	Pressure loss due to foil degradation	Target Solution: [^68^Zn]ZnO 1M in HNO_3_ 0.3M instead of 0.2M
^68^Ga not transferred from receiving vial Taddeo to Zr resin	Synthesis module: Vacuum pump failure due to evaporation of HNO_3_	Constant monitoring of the vacuum pump efficiency during the maintenance after production. If it fails the tests, replacement will be carried out
Eluition default of ^68^Ga activity trapped on resin	Manufacturing defect of the resin / HCl used for conditioning of the cartridge non-compliant	Preparation of new vials of HCL 4M
Low activity at EOB or at ^68^Ga-DOTATOC EOS	1. Worn target 2. HNO_3_ degradation (used for target solution and cleaning solution 3. Activity remains anchored into the cartridge and not eluted	1. Preventive maintenance of the ^68^Ga liquid target not beyond 3500 mA usually 2. Preparation of a new vial of cleaning solution (HNO_3_ 0.6M) using a new lot of HNO_3_ 70% 3. Preparation of a new vial of target solution ([^68^Zn]ZnO 1M in HNO_3_ 0.3M) using a new lot of HNO_3_ 70% 4. Preparation of new batch of reagents used during the purification of ^68^GaCl_3_
Other unforeseen issues	Cassette developer system: -Fluid loss from the cartridge -Issues during assembly of the cassette	1. Check the cartridges: if necessary, secure them using sealing film (Parafilm) 2. Qualified personnel through training, constant updates on any issues encountered and strategies for resolution. Check of the solution/cassette preparation by a second operator.

**Table 11:** Data relating to: EOB (MBq), EOS [^68^Ga]Ga-DOTATOC (MBq), Yield of synthesis (%) by Period 1 and 2.

**-**	**Period**	**Mean**	**Minimum**	**Maximum**
EOB (MBq)	1	3933	3552	4144
-	2	4107	3330	4366
EOS [^68^Ga]Ga-DOTA-TOC (MBq)	1	1506.3	1004	1893
-	2	1633.1	950	2514
Decay corrected yield (%)	1	46.0	28.9	57.6
-	2	49.8	34.4	68.3

**Table 12:** Independent samples student’s t-test (H_A_: m _1_/period 1< m _2_/period 2).

**-**	**-**	**Statistic**	**df**	***p***
EOB (MBq)	Student's t	-6.91ᵃ	286	< .001
EOS ^68^Ga DOTA-TOC (MBq)	Student's t	-3.27ᵃ	286	< .001
Yield (%)	Student's t	-1.51ᵃ	286	0.066

**Note: ** H_A_ μ _1_ < μ _2_; ᵃ Levene's test is significant (*p* < .05), suggesting a violation of the assumption of equal variances.

## Data Availability

The data that support the findings of this study are available from the corresponding author, [MC], on special request.

## LIST OF ABBREVIATIONS

^68^Ga: ^68^Gallium
cGRPP: Guidelines on Good Radiopharmacy Practice
EANM: European Association of Nuclear Medicine
EOB: End of Beam
EOS: End of Synthesis
GMP: Good Manufacturing Practices
HPLC: High Performance Liquid Chromatography
Ph. Eur: European Pharmacopoeia
SmPC: Summary of Product Characteristics
